# Supplementary material for: The eukaryotic bell-shaped temporal rate of DNA replication origin firing emanates from a balance between origin activation and passivation
Source: eLife. 2018 Jun 1;7:e35192. doi: 10.7554/eLife.35192 (PMC6033540; doi:10.7554/eLife.35192)
Supplement: Supplementary file 1. [file elife-35192-supp1.docx]

Supplementary information for:

## The eukaryotic bell-shaped temporal rate of DNA replication origin firing emanates from a balance between origin activation and passivation

by

Jean-Michel Arbona, Arach Goldar, Olivier Hyrien, Alain Arneodo, and Benjamin Audit

# Number of replica and parameters used for the simulations

Here we report the additional parameters (time step *dt* and number of replica) as well as the parameters detailed in the main text for each simulation series reported in the different figures.

## Figure 1

|  | N diffusing elements | N replica | fiber length | *kon* (/min) | periodic | ramp time | *ρ*0  (/kb) | time step |
| --- | --- | --- | --- | --- | --- | --- | --- | --- |
|  |  |  | (kb) |  |  | (min) |  | (min) |
| Fig. 1 blue | 1000 | 10000 | 3000 | 0.00005 | True | 3 | 0.30 | 0.1 |
| Fig. 1 green | 250 | 30000 | 3000 | 0.00060 | True | 3 | 0.50 | 0.1 |
| Fig. 1 red | 165 | 30000 | 3000 | 0.00600 | True | 3 | 0.28 | 0.1 |

- 1. **Figure 2(a)**

### Well-mixed model simulations

| N diffusing | N replica | fiber | *kon* (/min) | periodic | ramp | *ρ*0 | time |
| --- | --- | --- | --- | --- | --- | --- | --- |
| elements  Fig. 2(a) blue 187 | 3000 | length  (kb) 3000 | 0.003 | False | time  (min) 3 | (/kb)  0.70 | step  (min) 0.1 |
| Fig. 2(a) red 165 | 30000 | 3000 | 0.006 | True | 3 | 0.28 | 0.1 |

**3D model simulations**

N diffusing elements

N replica fiber

length (kb)

*kon* (/min) periodic ramp

time (min)

*ρ*0

(/kb)

time step (min)

Fig. 2(a) red squares 165 100 3000 0.007819 True 3 0.28 0.02

## Figure 2(b)

### Well-mixed simulations

N diffusing elements

*Np-ori* (0) N replica *kon* (/min) ramp time

(min)

time step (min)

total DNA length (kb)

Fig. 2(b) blue 143 410 2000 0.0036 10 0.2 12115

Fig. 2(b) yellow 143 626 2000 0.0036 10 0.2 12115

Fig. 2(b) purple 143 829 2000 0.0036 10 0.2 12115

### 3D model simulations

N diffusing elements

*Np-ori* (0) N replica *kon* (/min) ramp time

(min)

time step (min)

total DNA length (kb)

Fig. 2(a) purple squares

143 764 50 0.004561 10 0.02 12115

It is to be noted that the number of potential origins is different from 829. It is due to the coarse graining of the simulation, where origins of replication closer than 5 kb were merged.

## Figure 2(c)

|  | N diffusing elements | N replica | fiber length | *kon* (/min) | periodic | ramp time | *ρ*0  (/kb) | time step |
| --- | --- | --- | --- | --- | --- | --- | --- | --- |
|  |  |  | (kb) |  |  | (min) |  | (min) |
| 0 | 12 | 1000 | 3000 | 0.008 | True | 60 | 0.01 | 0.25 |
| 1 | 12 | 1000 | 3000 | 0.008 | True | 60 | 0.05 | 0.25 |
| 2 | 165 | 1000 | 3000 | 0.012 | True | 5 | 0.10 | 0.10 |
| 3 | 165 | 1000 | 3000 | 0.012 | True | 5 | 0.20 | 0.10 |
| 4 | 165 | 1000 | 3000 | 0.012 | True | 5 | 0.30 | 0.10 |
| 5 | 165 | 1000 | 3000 | 0.012 | True | 5 | 0.40 | 0.10 |
| 6 | 165 | 1000 | 3000 | 0.012 | True | 5 | 0.60 | 0.10 |
| 7 | 165 | 1000 | 3000 | 0.012 | True | 5 | 0.80 | 0.10 |
| 8 | 165 | 1000 | 3000 | 0.012 | True | 5 | 1.00 | 0.10 |
| 9 | 12 | 1000 | 3000 | 0.008 | False | 60 | 0.01 | 0.25 |
| 10 | 12 | 1000 | 3000 | 0.008 | False | 60 | 0.05 | 0.25 |
| 11 | 165 | 1000 | 3000 | 0.012 | False | 5 | 0.20 | 0.10 |
| 12 | 165 | 1000 | 3000 | 0.012 | False | 5 | 0.30 | 0.10 |
| 13 | 165 | 1000 | 3000 | 0.012 | False | 5 | 0.40 | 0.10 |
| 14 | 165 | 1000 | 3000 | 0.012 | False | 5 | 0.60 | 0.10 |
| 15 | 165 | 1000 | 3000 | 0.012 | False | 5 | 0.80 | 0.10 |
| 16 | 165 | 1000 | 3000 | 0.012 | False | 5 | 1.00 | 0.10 |

The simulations corresponding to the periodic *p-ori* distribution scenario (blue) are num- bered from 0 to 8, and are ordered by increasing values of the density *ρ*0. The simulations corresponding to the uniform *p-ori* distribution scenario (green) are numbered from 9 to 16 and are ordered by increasing values of the density *ρ*0.

# List of symbols

- *Nfired*(*t, t* + *dt*) : number of origins that have fired between *t* and *t* + *dt*.
- *NF D* (*t*) : number of free firing factors at time *t*.

*∗* *v*

- *N*

=

*F D kond*(*t*)

: critical number of firing factors.

- *Np-ori* (*t*) : number of potential origins at time *t*.
- *ρp-ori* (*t*) : density of origins in unreplicated DNA at time *t*.
- *kon* : reaction constant between origins and firing factors.
- *d*(*t*) : mean distance between unreplicated *p-oris* at time *t*.
- *v* : fork speed.
- *I*(*t*) : firing rate per length of unreplicated DNA.
- *Lunrep*(*t*) : length of unreplicated DNA.
- *NT*

*D*

: total number of firing factors.

- *L* : total length of DNA.

# References

[1] G. M. Alvino, D. Collingwood, J. M. Murphy, J. Delrow, B. J. Brewer, and M. K. Raghura- man. Replication in hydroxyurea: It’s a matter of time. *Mol. Cell. Biol.*, 27(18):6396–6406, 2007.
